# Microarray Analyses and Comparisons of Upper or Lower Flanks of Rice Shoot Base Preceding Gravitropic Bending

**DOI:** 10.1371/journal.pone.0074646

**Published:** 2013-09-05

**Authors:** Liwei Hu, Zhiling Mei, Aiping Zang, Haiying Chen, Xianying Dou, Jing Jin, Weiming Cai

**Affiliations:** Institute of Plant Physiology and Ecology, Shanghai Institutes for Biological Sciences, Graduate School of Chinese Academy of Sciences, Chinese Academy of Sciences, Shanghai, China; Wake Forest University, United States of America

## Abstract

Gravitropism is a complex process involving a series of physiological pathways. Despite ongoing research, gravitropism sensing and response mechanisms are not well understood. To identify the key transcripts and corresponding pathways in gravitropism, a whole-genome microarray approach was used to analyze transcript abundance in the shoot base of rice (*Oryza sativa* sp. japonica) at 0.5 h and 6 h after gravistimulation by horizontal reorientation. Between upper and lower flanks of the shoot base, 167 transcripts at 0.5 h and 1202 transcripts at 6 h were discovered to be significantly different in abundance by 2-fold. Among these transcripts, 48 were found to be changed both at 0.5 h and 6 h, while 119 transcripts were only changed at 0.5 h and 1154 transcripts were changed at 6 h in association with gravitropism. MapMan and PageMan analyses were used to identify transcripts significantly changed in abundance. The asymmetric regulation of transcripts related to phytohormones, signaling, RNA transcription, metabolism and cell wall-related categories between upper and lower flanks were demonstrated. Potential roles of the identified transcripts in gravitropism are discussed. Our results suggest that the induction of asymmetrical transcription, likely as a consequence of gravitropic reorientation, precedes gravitropic bending in the rice shoot base.

## Introduction

Gravitropism is the directed growth of a plant or plant organ in response to gravity, which can be divided into a series of events: perception, transduction and response [1], [2]. Gravity is known to stimulate different growth rates on opposite flanks of the root and shoot, causing bending or curvature of the plant organ in the process of gravitropism [3], [4].

The statolith theory of gravity perception is widely accepted and suggests that perception occurs by reorientation of starch grains (statoliths) in specialized plant cells called statocytes [5], [6]. The gravistimulating signal is transferred by the cytoskeleton, actin-based cytoskeletal network and plasma membrane receptors [7], [8], [9]. Inositol-1,4,5-trisphosphate (IP3), pH and Ca^2+^ are second messengers of early signal events in the transduction of physical alterations into a biochemical signal within the plant cell [10], [11], [12].

Gravistimulation signals are transmitted intercellularly by lateral asymmetric transport of auxin [13], [14]. Besides auxin, ethylene [15], [16], [17], cytokinin [18], [19], brassinosteroids (BR) [20] and gibberellic acid (GA) [21], [22], [23] also play a role in gravitropism. Whole genome transcriptomic analysis can be used to discover crucial factors controlling the gravity signal transduction cascade and elucidate mechanisms of the plant gravitropic response at the molecular level [24], [25], [26]. Gravitropic bending results from the asymmetric growth between the lower and upper half flanks of the responsive parts of the plant. While the genes involved in the asymmetrical growth of gravitropic bending are not fully known, a transcriptional gradient has been reported to precede growth responses involved in the gravitropic bending [21], [27]. Our previous study showed that gravity induces asymmetric accumulation of indole-3-acetic acid (IAA), the major plant auxin at the bending site prior to upward bending of shoot base after gravistimulation, with higher IAA in the lower flank than the upper flank of rice shoot base [28].

MapMan is a user-driven tool that displays large data sets onto diagrams of metabolic pathways or other processes, which enables statistical evaluation of differences in response of sets of genes assigned to different biological functions and allows more validated interpretation of global cellular responses [29], [30]. PageMan is often used together with MapMan, which allows genome-level responses to be compared across several microarray experiments [31]. To understand the significance of these distinct patterns of transcript abundance, MapMan and PageMan analyses were conducted in this study, and each provided a different insight into the molecular understanding of gravitropism in rice. Diagrams generated by MapMan analysis allows the visualization of changes in expression of individual genes grouped in “BINs” by function or class. This type of analysis was performed by considering only significantly up- or down-regulated functional gene categories and allowed construction of relevant pathways in rice gravitropism. Our data provided a spatial transcript abundance profile of the gravitropic bending site on the whole-genome scale, which revealed asymmetric regulations of transcripts between upper and lower flanks in the phytohormone, signaling, RNA transcription, metabolism and cell wall-related categories. While most of the pathways have been reported previously, several new transcripts asymmetrically expressed between the upper and lower flanks of the shoot base after gravistimulation were identified, which may be key factors for understanding the mechanism of gravitropism.

## Results

### Transcriptional changes in the upper and lower flanks of rice shoot base

**Figure 1:**
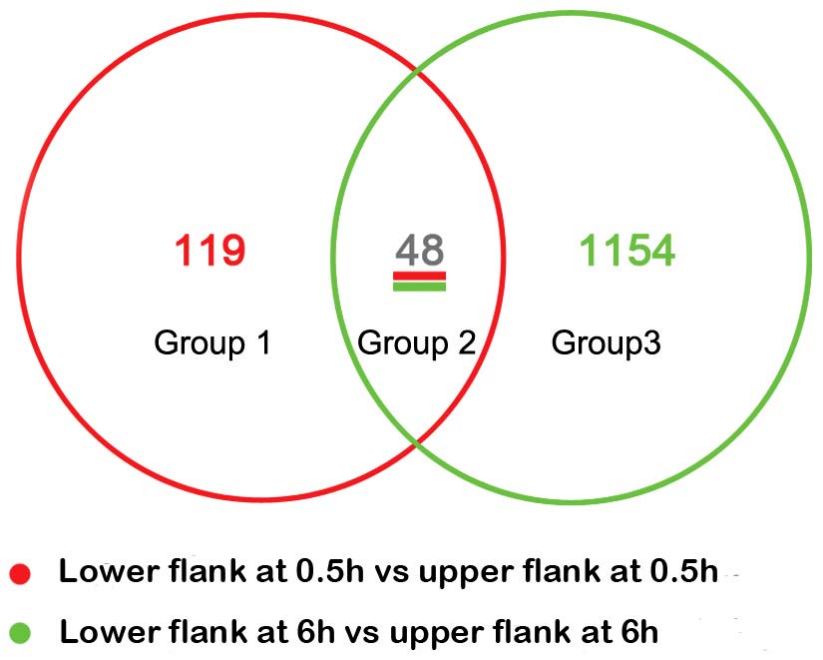
Venn diagrams constructed based on significantly different transcript abundance in gravistimulated rice shoot base. Venn diagrams show the distribution of transcripts that were significantly changed in abundance by at least 2-fold (*P* value <0.05) between the lower flank and upper flank of the rice shoot base at 0.5 h and 6 h after gravitropic stimulation.

Rice seedlings were gravitropically stimulated for 0.5 h and 6 h, and mRNA isolated from opposing flanks of the shoot base were used as probes in whole genome rice GeneChip (Affymetrix) microarrays to order to identify key transcripts involved in the gravitropic response. Three replicates were used to minimize the biological variance. To reduce potential contamination and error from sampling, RNA was extracted from 50 non-treated rice shoot bases (0 h) and 100 lower or upper flanks of shoot bases from horizontal reorientated rice seedlings after 0.5 h (upper and lower) or 6 h (upper and lower) of gravitropic stimulation. Microarray analyses were repeated with three biological replicates for each time point. The Affymatrix GeneChip of Rice Genome Array contains probe sets to detect transcripts from the entire rice genome, including 55,515 transcripts, between 46.7–49.6% of which were detectable in 15 microarray hybridizations. By comparing datasets between upper and lower samples, 167 and 1,202 transcripts were found to be differentially expressed by at least 2-fold at 0.5 h and 6 h, respectively (Fig. 1). Venn diagrams were constructed to identify transcripts that exhibited the same gravity-induced behavior in the rice shoot base. The comparison was performed for transcripts that were significantly different in abundance between the two half flanks (Fig. 1). Forty-eight transcripts were found to be changed both at 0.5 h and 6 h (Table S1); meanwhile, 119 transcripts were only altered at 0.5 h (Fig. S4, Table S2), and 1154 transcripts were changed at 6 h (Fig. S5, Table S3).

### Functional classification of significantly changed transcripts during gravitropism

PageMan analysis revealed that a variety of cellular processes were affected during the gravitropism process and confirmed that a number of transcripts were significantly changed between the upper and lower half flanks of the rice shoot base at 0.5 h and 6 h after horizontal reorientation (Fig. S1). In comparing the abundance of transcripts in the upper half flank with that in the lower half flank at 0.5 h and 6 h, the transcripts with higher abundance in the lower half flank included those in the photosynthesis (PS) and DNA categories, while those of glycolysis, fermentation, lipid metabolism, misc and cell organization were repressed. The results also showed differences in transcriptional regulation within other categories between 0.5 h and 6 h. For example, gene transcripts involved in metabolism of phenylpropanoids and flavonoids were more abundant in the lower flank at 0.5 h, but only that of phenylalanine ammonia-lyase PAL (Os02g41650), which is involved in phenylpropanoid metabolism, was less abundant in the lower half flank at 6 h. Many transcripts in the categories of “cell wall” and “hormone metabolism” were more abundant at 6 h, and a few transcripts encoding cell wall proteins were less abundant at 0.5 h. Most of the regulated transcripts involved in signaling (e.g. receptor kinase signaling pathway and phosphinositides signaling pathway) were lower in abundance in the lower flank at both 0.5 h and 6 h.

In order to study the sequential changes in transcription related to gravitropism, samples harvested at 0.5 h and 6 h after gravitropic stimulation both from the upper and lower flanks were compared with the non-treated control sample (Fig. S2). MapMan and PageMan analyses revealed that some transcripts at 0.5 h and 6 h were not different in abundance between the lower half and upper half flanks, but they were significantly different from that of the control. The comparisons between 0 h and lower/upper flanks at 0.5 h showed that transcripts in the categories of cell wall, sulfur metabolism, nucleotide metabolism, protein synthesis and targeting, and transport of amino acid were up-regulated, while those related to amino acid metabolism, minor carbohydrate metabolism, protein degradation and abiotic stress were less abundant with no difference between the two flanks. In addition, the comparison between samples at 0 h and upper/lower half flanks at 6 h showed that the transcripts of genes encoding GAP, fructose-1,6-bisphosphatase (FBPase) and ribulose-1,5-bisphosphate carboxylase oxygenase (RuBisCO), trehalase and PAL were more abundance in both flanks. Thus, transcripts involved in the degradation of the branched chain group in amino acid metabolism and minor carbohydrate metabolism were more abundant in both flanks at 6 h, with no difference between the two flanks. Changes of these transcripts may be the result of combined effects of the gravitropic stimulus and circadian rhythms.

### Transcriptional changes of secondary metabolism and inositol synthesis in gravitropism

Flavonoids (or bioflavonoids) and phenylpropanoids are important secondary metabolites. Flavonoids have been reported to influence auxin transport and alter the response of *Arabidopsis* roots to gravity [16], [17], [32]. Here, our data showed that the transcripts encoding PAL and flavonol synthase (FLS, Os10g40934) were induced in the lower flank at 0.5 h, which may contribute to the synthesis of quertecin kaempferol and myricetin and regulate the transport of auxin in gravitropism. Interestingly, the transcript encoding the inositol synthesis related inositol monophosphatase 3 (Os03g39000) was induced in the lower flank at both 0.5 and 6 h.

### Changes of RNA transcripts and regulation of protein synthesis and degradation in gravitropism

Most gene transcripts involved in the regulation of transcription (e.g., Alfin, CPP1-related transcription factor, E2F/DP transcription factor, G2 like, SBP, triple-helix transcription factor, WRKY domain transcription factor, bZIP family transcription factor, ATSR transcription factor, chromatin transcription factor, global transcription factor, JUMONJI family, methyl domain binding domain protein, PHD finger transcription factor and SET domain transcriptional regulator) were less abundant in the lower flanks at 0.5 h and 6 h. Increased abundance was detected for the transcripts of the AS2 transcription factor and the AUX/IAA family. In addition, our data showed that gene transcripts related to RNA processing, including RNA splicing and RNA binding, were less abundant in the lower flank both at 0.5 h and 6 h. The transcript for RNA helicase was less abundant in the lower flank at 0.5 h. The transcripts of genes coding for proteins targeting to the secretory pathway were more abundant in the lower flank at 6 h, and those of proteins related to degradation, such as ubiquitin, ubiquitin E3, ubiquitin E3 RING, ubiquitin E3 SCF, ubiquitin E3 SCF F-box and ubiquitin protease, were less abundant in the lower flank at 6 h (Fig. S1).

### Changes in abundance and temporal sequence of transcripts involved in hormonal regulation in gravitropism

**Figure 2:**
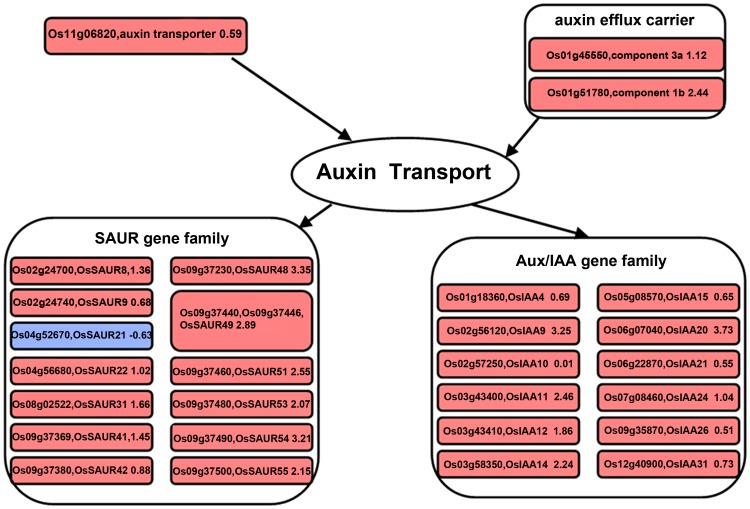
Changes of transcripts in the auxin related pathway at 6 h after gravistimulation. Visualization of modulated gene expression in the auxin pathway: lower flank at 6 h vs. upper flank at 6 h. Significant fold changes in transcript abundance were log transformed and represented as up-regulated (red square) or down-regulated (blue square).

It is well known that efflux carriers and transporters affect the lateral relocation of auxin [13], [14], and auxin flow in the root cortex is important for optimal gravitropic response. Gravitropism depends on the polar deployment of the PIN-formed auxin efflux carrier PIN2 [33]. In our results, the greater abundance of transcripts for the auxin efflux carrier (orthologs of PIN4 and PIN8) and auxin transporter (orthologs of the auxin influx carrier LAX3 in *Arabidopsis*) in the lower flank at 6 h may have contributed to the transport of auxin between the upper and the lower flanks (Fig. 2), and caused the faster growth of cells at the lower flank of the shoot base after gravistimulation. Small auxin up-regulated RNA (*SAUR*) and *AUX/IAA* family genes are quick auxin-responsive genes, and several *SAUR* genes have been found to be highly expressed in tissues undergoing differential cell expansion such as during tropic growth [27], [33], [34]. It has been reported that GFP–*SAUR19* transgenic *Arabidopsis* plants exhibit an altered IAA response and transport in roots [35]. In our results, all of the auxin-responsive gene transcripts (except one), including *SAUR* and *AUX/IAA* family genes, were more abundant in the lower flank than the upper flank of the shoot base at 6 h (Fig. 2), which may regulate the asymmetrical distribution of auxin and expression of downstream genes in gravitropism.

**Figure 3:**
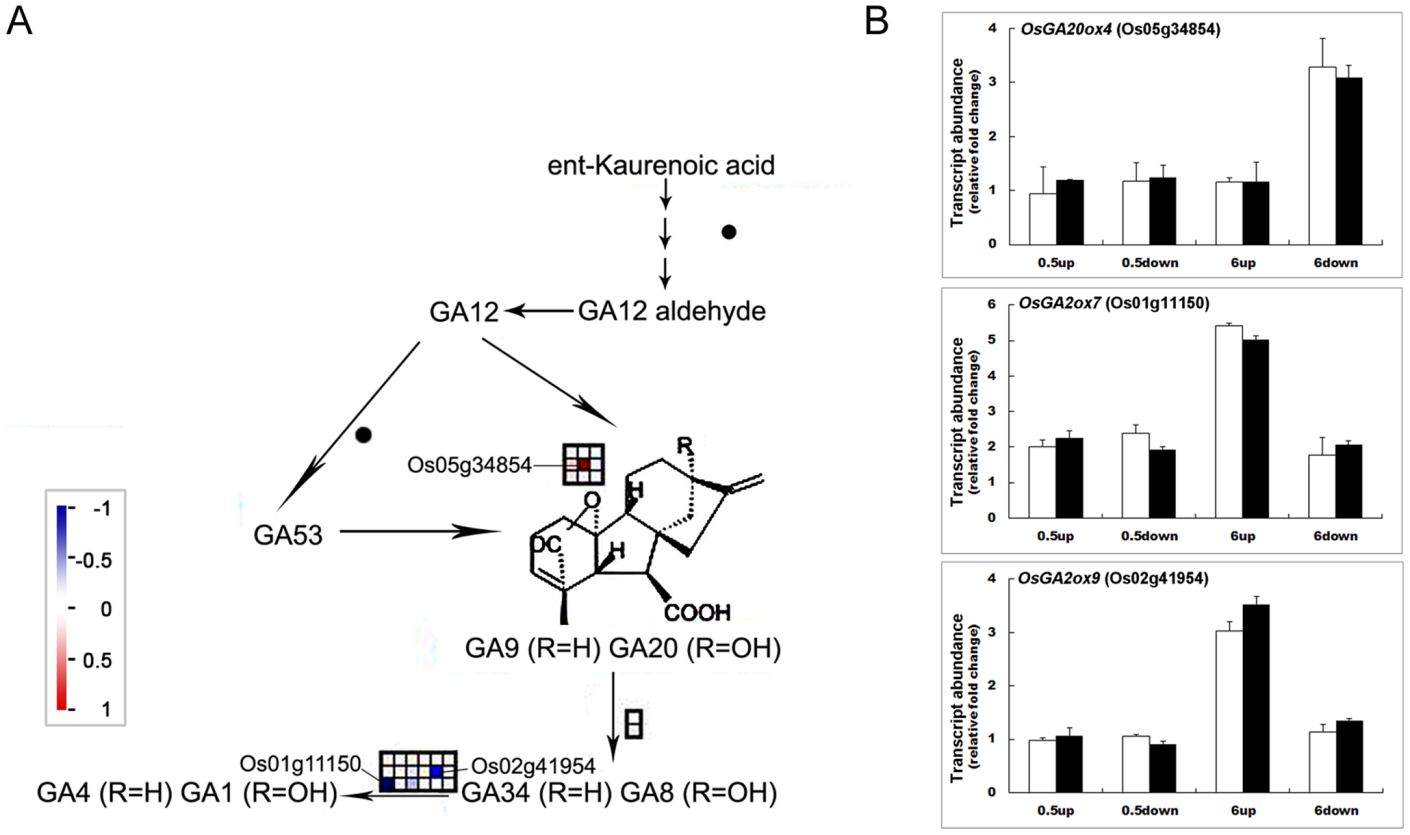
Changes of transcripts for GA metabolism at 6 h after gravistimulation. A. MapMan output was used to illustrate the significant transcriptional changes across the tissue in GA metabolism at 6 h after reorientation. The reported values are log transformed ratios of transcripts in the lower flank to that in the upper flank. Each transcript is indicated as up-regulated (red square; increase in the ratio of the lower to upper transcript values) or down-regulated (blue square; decrease in the ratio of the lower to upper transcript values). Squares arranged in rows and columns represent individual transcripts at a single time point in the process. An individual square in a given area has the same color as the guide bar in the same column. A filled circle indicates that no transcript was detected. B. qRT-PCR analysis of *OsGA20ox4* (Os05g34854), *OsGA2ox7* (Os01g11150) and *OsGA2ox9* (Os02g41954) expression after horizontal reorientation. Relative changes in transcript abundance after gravity stimulation compared to the vertical control at each time point were analyzed by qRT-PCR, and results were compared with the microarray data. Relative changes in transcript abundance of qRT-PCR were all determined using the non-reoriented control at the relevant time point as reference. 0.5 h down, 0.5 h up, 6 h down and 6 h up represent for lower flank at 0.5 h, upper flank at 0.5 h, lower flank at 6 h and upper flank at 6 h, respectively. The relative gene transcript abundance was calculated as the ratio between the control and gravistimulated samples at each time point. Values are means ± SD; n = 3; □ real-time PCR results; ▪ microarray data.

GA metabolism has been reported to play a role in gravitropism [21], [36]. Increased content of GA_1/3_ (belonging to bioactive GAs) was observed in the lower flank of rice shoot base after gravistimulation [1], implicating the involvement of GA in gravitropism. In our study, we found a greater transcript abundance of *OsGA20ox4* (Os05g34854) [37] and lower transcript abundance of *OsGA2ox7* and *OsGA2ox9* (Os01g11150 and Os02g41954, respectively) in the lower flank [38], which is responsible for irreversible deactivation of GAs by 2-hydroxylation (Fig. 3A). The results were verified by quantitative real-time PCR (qRT-PCR) (Fig. 3B). We predicted that differential expression of *GA2ox* and *GA20ox* may have caused asymmetric accumulation of bioactive GAs between the lower and upper flanks of the shoot base via deactivating more active GAs in the upper flank.

**Figure 4:**
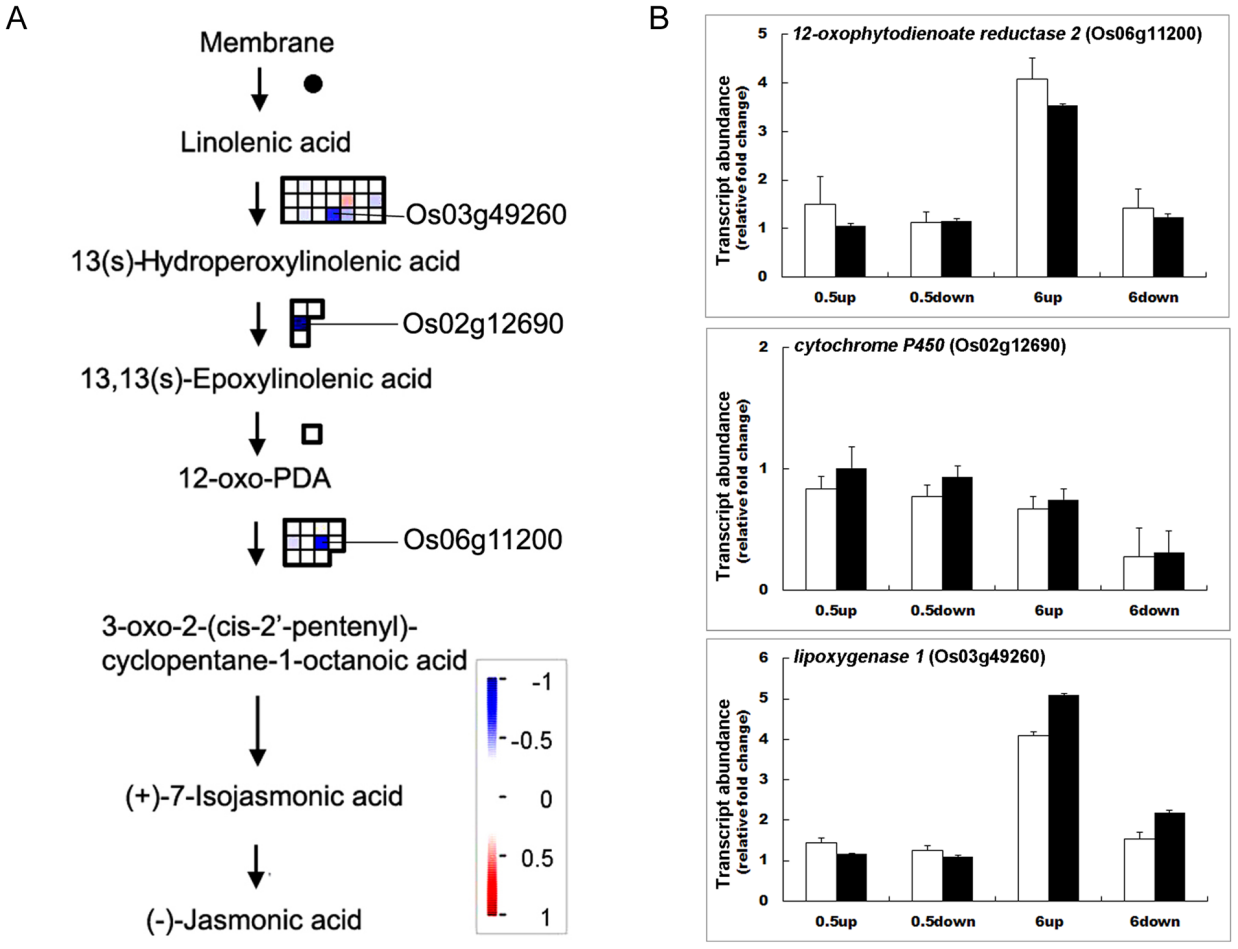
Changes of transcripts for JA metabolism at 6 h after gravistimulation. A. MapMan output was used to illustrate significant transcriptional changes across the tissue on JA metabolism at 6 h after reorientation. The reported values are log transformed ratios of transcripts in the lower flank to the upper flank. Each transcript is indicated as being up-regulated (red square; increase in the ratio of the lower to upper transcript values) or down-regulated (blue square; decrease in ratio of the lower to upper transcript values). Squares arranged in rows and columns represent individual transcripts at a single time point in the process. An individual square in a given area has the same color as the guide bar in the same column. A filled circle indicates that no transcript was detected in the process. B. qRT-PCR analysis of *lipoxygenase 1* (Os03g49260), *cytochrome P450* (Os02g12690) and *12-oxophytodienoate reductase 2* (Os06g11200) expression after horizontal reorientation. Relative changes in transcript abundance after gravity stimulation compared to the vertical control at each time point were analyzed by qRT-PCR, and results were compared with the microarray data. The relative gene transcript abundance was calculated as the ratio between the control sample and gravistimulated sample at each time point. Values are means ± SD; n = 3; □ real-time PCR results; ▪ microarray data.

Gutjahr *et al.* (2005) detected a gradient of jasmonic acid (JA) opposing the auxin gradient related to gravitropism in rice [39]. Here, our data showed that transcripts of three genes encoding lipoxygenase 1 (Os03g49260), cytochrome P450 (Os02g12690) and 12-oxophytodienoate reductase 2 (Os06g11200), which are involved in the synthesis of JA, showed higher levels in the upper flank at 6 h (Fig. 4A). These results were verified by qRT-PCR (Fig. 4B). Thus, products of the up-regulated genes may contribute to formation of the JA gradient at the shoot base of rice during gravitropism.

BRs have been reported to stimulate plant tropism through modulation of polar auxin transport and BR function as a negative regulator of the shoot graviresponse in *Arabidopsis* [20], [40]. Here, two transcripts related to BR metabolism were found to be involved in gravitropism. *CYP734A6* (Os01g29150), which encodes cytochrome P450 and functions in BR inactivation [41], was specifically expressed in the lower flank of the rice shoot base, whereas another cytochrome P450, encoded by *CYP90A1/OsCPD2* (Os12g04480) and functions in BR biosynthesis [42], [43], [44], was more strongly expressed than in the upper flank.

In addition, cytokinin synthesis-related gene transcripts were less abundant in the lower flank at 0.5 h, while ethylene-related transcripts were more abundant in the lower flank (Fig. S3). These transcripts may also be involved in the modulation of flavonoid accumulation in gravitropism [16], [18].

### Changes in transcripts related to cell wall loosening in gravitropism

**Figure 5:**
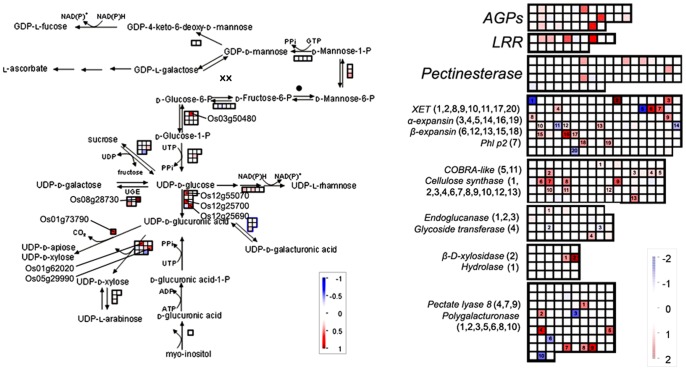
Changes of transcripts for cell wall precursors and cell wall protein related pathways between lower and upper half flanks at 6 h after gravistimulation. MapMan output was used to illustrate the significant transcriptional changes across the tissue of cell wall precursors and cell wall proteins at 6 h after reorientation. The reported values are log transformed ratios of transcripts in the lower flank to the upper flank. Each transcript is indicated as being up-regulated (red square; increase in the ratio of the lower to upper transcript values) or down-regulated (blue square; decrease in ratio of the lower to upper transcript values). Squares arranged in rows and columns represent individual transcripts at a single time point in the process. The individual square in a given area has the same color as the guide bar in the same column. A filled circle indicates that no transcript was detected in the process. Significantly changed transcripts in each process were marked with a series of numbers.

Cell wall composition and extensibility change during gravitropism [45]. A previous microarray study showed that cell wall-related transcripts are regulated in gravitropism [46]. Here, our data showed asymmetrical regulation of the cell wall precursors and cell wall loosening related transcripts in the gravitropic bending of rice shoot bases (Fig. 5). Gene transcripts of pectin esterase, xylosidase, hydrolase, cellulase and pectate lyase were more abundant in the lower flank at 6 h. Transcripts of multiple expansins (α-expansin and β-expansin) and xyloglucan endotransglycosylase coding genes were more abundant in the lower flank at 6 h. In addition, cell wall precursor-related transcripts were more abundant in the lower flank at 6 h, including those encoding bifunctional polymyxin resistance arnA protein (Os01g73790), which plays a role in the synthesis of UDP-D-apiose and UDP-D-xylose; UDP-D-galactose synthesis-related NAD dependent epimerase (Os08g28730); UDP-glucuronic acid decarboxylase (Os05g29990 and Os01g62020), which are involved in the synthesis of UDP-D-xylose; UDP-glucose 6-dehydrogenase (Os12g55070, Os12g25700 and Os12g25690), which is responsible for the synthesis of UDP-D-glucuronic acid and synthesis-related phosphoglucomutase (Os03g50480). The coordinated increase in levels of these cell wall loosening and cell wall precursors synthesis-related transcripts in the lower flank may contribute to gravitropism by affecting cell wall relaxation and extension.

### Changes of transcripts related to glycolysis and tricarboxylic acid (TCA/citric) cycle in gravitropism

**Figure 6:**
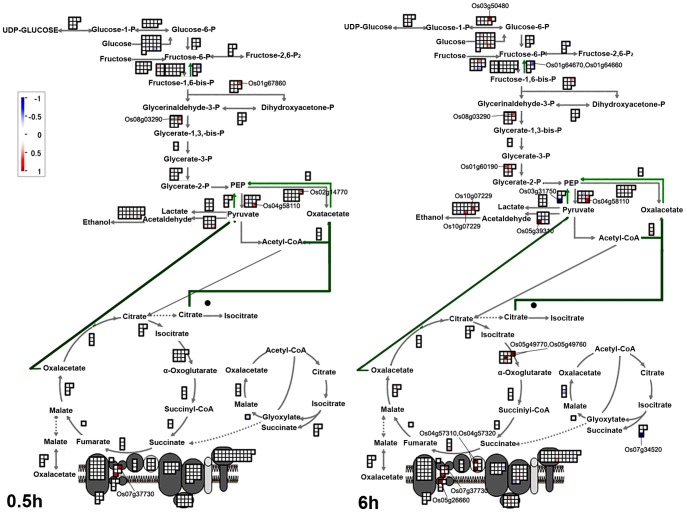
Changes of transcripts for glycolysis and the TCA cycle at 0.5 h and 6 h after gravistimulation. MapMan output was used to illustrate the significant changes across the tissue on glycolysis and the TCA cycle at 0.5 h and 6 h after reorientation. Reported values are log transformed ratios of transcripts in the lower flank to the upper flank. Each transcript is indicated as being up-regulated (red square; increase in the ratio of the lower to upper transcript values) or down-regulated (blue square; decrease in ratio of the lower to upper transcript values). Squares arranged in rows and columns represent individual transcripts at a single time point in the process. An individual square in a given area has the same color as the guide bar in the same column. A filled circle indicates that no transcript was detected in the process.

Both glycolysis and the citric acid cycle are important metabolic pathways which generate usable energy. Our data showed that the greater abundance of transcripts encoding glyceraldehyde-3-phosphate dehydrogenase (Os08g03290), pyruvate kinase (Os04g58110), phosphoenolpyruvate carboxylase 1 (Os02g14770), ATP synthesis-related rotenone-insensitive NADH-ubiquinone oxidoreductase (Os07g37730) and fructose-bisphosphate aldolase cytoplasmic isozyme (Os01g67860) in the lower flank at 0.5 h may provide the energy for gravitropic bending by contributing to the generation of ATP (Fig. 6). Many transcripts coding for glycolysis-related and TCA-related proteins showed different levels of abundance between the lower and upper flanks of the rice shoot base at 6 h. The more abundant transcripts included phosphoglucomutase (Os03g50480), glyceraldehyde-3-phosphate dehydrogenase (Os08g03290), 2,3-bisphosphoglycerate-independent phosphoglycerate mutase (Os01g60190), pyruvate kinase (Os04g58110), pyruvate decarboxylase (Os05g39310) and alcohol dehydrogenase 2 (Os10g07229); meanwhile, pyrophosphatase (Os01g64670 and Os01g64660), fructose-1,6-bisphosphatase (Os01g64660) and pyruvate phosphate dikinase (Os03g31750) were among the less abundant transcripts.

The more abundant transcripts of CTP synthase (Os05g49770) and isocitrate dehydrogenase (Os05g49760) in the lower flank and less abundant transcript of isocitrate lyase (Os07g34520) in the lower flank may contribute to the TCA process. Several transcripts encoding mitochondrial electron transport/ATP synthesis proteins were more abundant in the lower flank at 6 h, including rotenone-insensitive NADH-ubiquinone oxidoreductase (Os07g37730 and Os05g26660), thiol-disulphide oxidoreductase (Os04g57310) and immutans protein (Os04g57320). These results suggested that the acceleration of glycolysis and the TCA cycle may provide energy for gravitropic bending.

### Changes of transcripts for sucrose-starch metabolism in gravitropism

**Figure 7:**
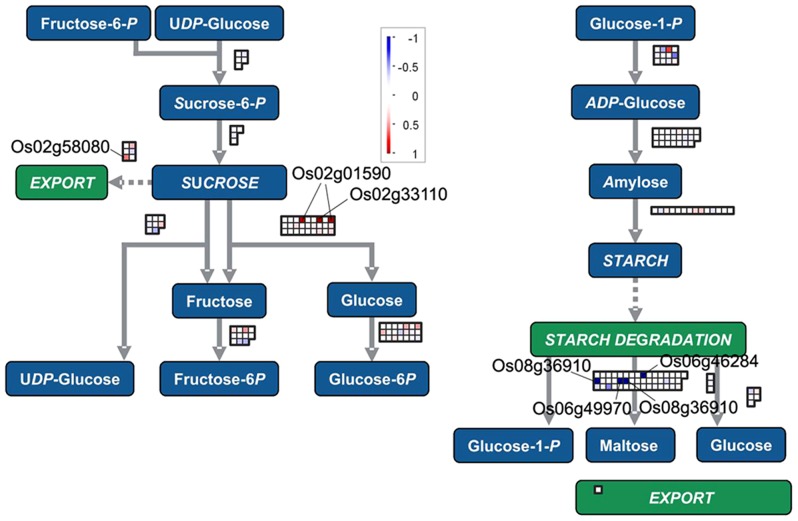
Changes of transcripts for sucrose-starch metabolism at 6 h after gravistimulation. MapMan output was used to illustrate significant transcriptional changes across the tissue in cell wall precursors and cell wall proteins at 6 h after reorientation. The reported values were log transformed ratios of transcripts in the lower flank to the upper flank. Each transcript is indicated as being up-regulated (red square; increase in the ratio of the lower to upper transcript values) or down-regulated (blue square; decrease in the ratio of the lower to upper transcript values). Squares arranged in rows and columns represent individual transcripts at a single time point in the process. An individual square in a given area has the same color as the guide bar in the same column. A filled circle indicates that no transcript was detected in the process.

Our previous studies had demonstrated that hexoses including glucose are increased in the lower flank of the rice shoot base during gravitropic bending [28]. In this study, the transcript encoding the sucrose transport protein SUC3 (Os02g58080) was higher in the lower flank at 6 h. Transcript abundance of beta-fructofuranosidase (Os02g01590 and Os02g33110) was 2-fold and 2.2-fold higher, respectively, in the lower flank than that in the upper flank. By contrast, the starch degradation reaction seemed to be inhibited in the lower flank at 6 h, with several transcripts encoding starch degradation-related enzymes, such as alpha-glucosidase precursor (Os06g46284), alpha-amylase isozyme C2 precursor (Os06g49970), alpha-amylase isozyme 3D (Os08g36910), alpha-amylase isozyme 3D (Os08g36910) and beta-amylase (Os02g03690) which were less abundant by 0.37, 0.41, 0.45, 0.46 and 0.69-fold, respectively (Fig. 7). These data suggested that changes of transcripts related to sugar transport and metabolism may contribute to the difference in sugar content between the upper and lower parts relative to the bending site during the gravitropic response.

### Other significantly regulated transcripts in gravistimulated rice

In addition, we observed alterations in levels of transcripts involved in signaling, DNA regulation and various cellular processes at 0.5 h and 6 h. Plant glutathione S-transferases (GSTs) were suggested to be related to plant gravitropism [24], [47]. Moseyko (2005) found that GST belongs to the oxidative burst plant defense group, which is the largest functional category of gravity-regulated transcripts in *Arabidopsis*, but the gravitropic functions of those corresponding transcripts in rice are not clear.

### Validation of microarray data by qRT-PCR

**Figure 8:**
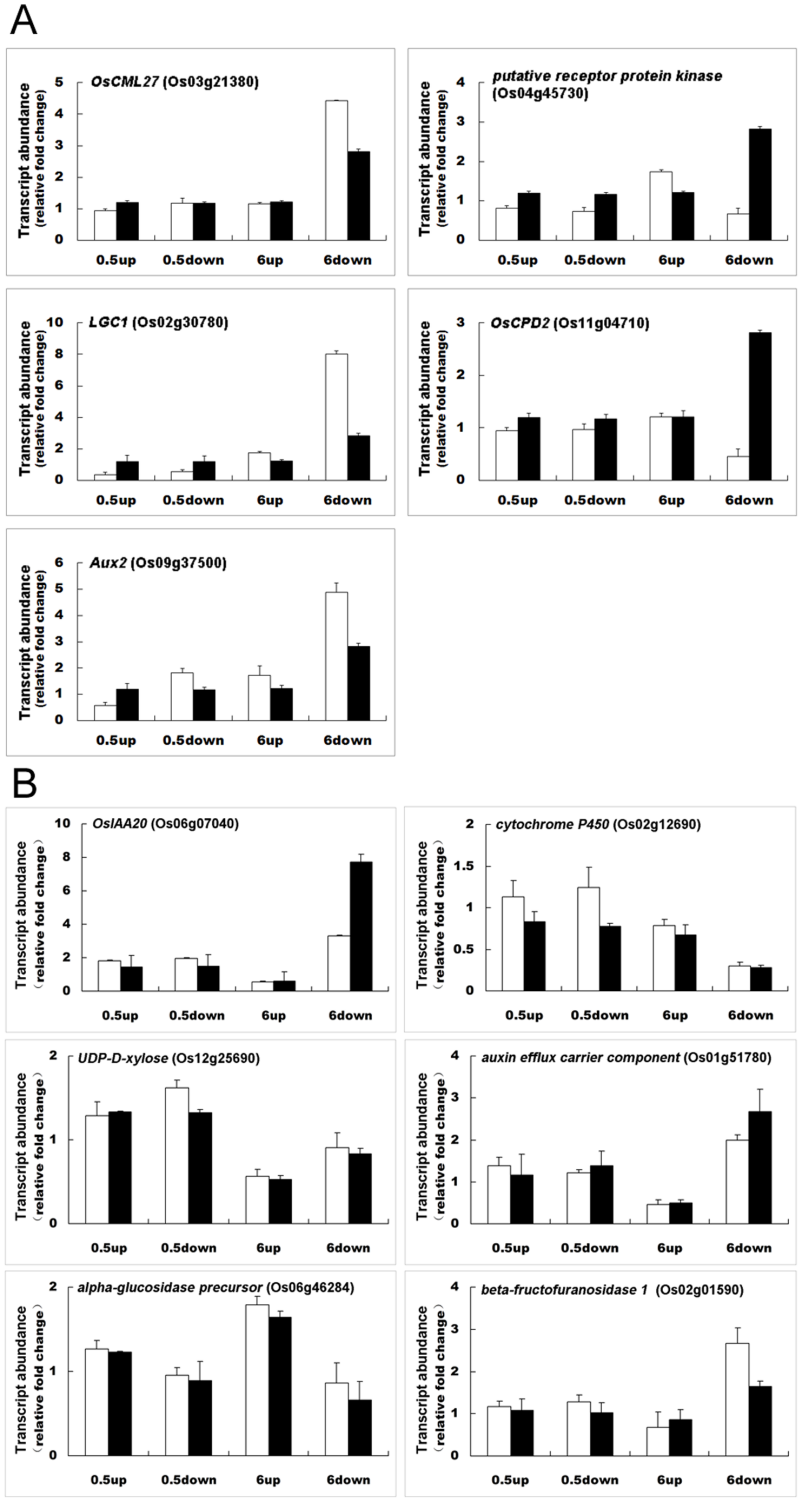
qRT-PCR validation of microarray analysis results. Relative changes in transcript abundance after gravity stimulation compared to the vertical control were analyzed by the microarray assay, and relative changes in transcript abundance observed by qRT-PCR all utilized the non-reoriented control at the relevant time point as a reference. A. qRT-PCR analysis of *OsCML27* (Os03g21380), *OsCPD2* (Os11g04710), a transcript encoding a putative receptor protein kinase (Os04g45730), *Aux2* (Os09g37500) and *LGC1* (Os02g30780) expression. B. qRT-PCR analysis of *OsIAA20* (Os06g07040), and coding genes for cytochrome P450 (Os02g12690), UDP-D-xylose (Os12g25690), auxin efflux carrier component (Os01g51780), α-glucosidase precursor (Os06g46284), β-fructofuranosidase 1 (Os02g01590). 0.5 h down, 0.5 h up, 6 h down and 6 h up represent for lower flank at 0.5 h, upper flank at 0.5 h, lower flank at 6 h and upper flank at 6 h, respectively. The relative mRNA abundance was calculated as the ratio between the control sample and gravistimulated sample at each time point. Values are means ± SD; n = 3; □ real-time PCR results; ▪ microarray data.

To validate the microarray data, five transcripts from the whole microarray dataset and six transcripts from the MapMan and PageMan results, which were significantly different in abundance between the lower and upper flanks, were randomly selected to be analyzed by qRT-PCR using three independent biological replicates. These gene transcripts included *OsCML27* (Os03g21380), *OsCPD2* (Os11g04710), a putative receptor protein kinase coding gene (Os04g45730), *Aux2* (Os09g37500) and *LGC1* (Os02g30780). The expression tendencies of these transcripts (either up-regulation or down-regulation) from microarray and qRT-PCR analyses were compared side-by-side at 0.5 h and 6 h (Fig. 8A). The relative abundance and the temporal changes in transcript levels after gravity stimulation were confirmed by qRT-PCR for transcripts of OsIAA20 (Os06g07040), cytochrome P450 (Os02g12690), UDP-D-xylose (Os12g25690), auxin efflux carrier component (Os01g51780), alpha-glucosidase precursor (Os06g46284) and beta-fructofuranosidase (Os02g01590), which were selected from the genes derived from results of MapMan and PageMan analyses. Compared with the upper half flank as a control, the up-regulated changes in the lower half flank at 6 h included transcripts of OsIAA20 (Os06g07040), UDP-glucose 6-dehydrogenase (Os12g25690), auxin efflux carrier component (Os01g51780) and beta-fructofuranosidase 1 (Os02g01590), while the down-regulated transcripts in the lower half flank at 6 h included those encoding cytochrome P450 (Os02g12690) and alpha-glucosidase precursor (Os06g46284). The results revealed that the expression tendencies were similar between qRT-PCR and microarray results for the transcripts selected from the PageMan and MapMan analyses (Fig. 8B).

## Discussion

Gravitropism, or bending of plants in response to gravity, is caused by differential growth rates between the two flanks of a responsive organ, which requires a complex signal transduction cascade. IP3, pH, and Ca^2+^ are second messengers of early signaling events in the transduction of physical alterations into a biochemical signal within the plant cell [10], [11], which may regulate specific gene expression to result in differential growth rates between two regions, causing bending or curvature of the plant organ. However, associations between all of these signal transduction events and the mechanism of asymmetric growth still require clarification. Whole genome expression analysis using gene chips makes identifying multiple regulatory genes on each side of a responsive organ during the gravitropic response technically possible. Thus far, only a few studies have reported transcriptome profiling of gravitropism, in which whole seedlings or specific segments of *Arabidopsis* roots were collected as experimental materials [24], [46]. In such cases, important genes which show asymmetrical transcript abundance between two flanks of the responding organs under gravistimulation and contribute to the gravitropic response may be missed, especially since it is difficult to separate the two flanks due to the small size of the *Arabidopsis* plant. Here we used 3-week-old rice seedlings as experimental materials, and the two flanks of the gravitropic responsive organ (i.e., rice shoot base) were fairly easy to separate [21]. By comparing transcript abundance in the lower and upper half flanks of the shoot base, genes responsive to gravistimulation were identified, although some of them which were expressed differentially at various timepoint may be also have been affected by the circadian rhythm. A total of 5,575 and 5,047 transcripts were detected in the upper and lower flanks of the shoot base, respectively. There were 167 and 1,202 significantly (*P*<0.05) transcripts that were altered by greater than 2-fold between the upper and lower flanks of the shoot base at 0.5 h and at 6 h, respectively. To our knowledge, this is the first study outlining the transcriptional network of negative gravitropism in the shoot base of rice. The analysis of distinct transcript levels in response to gravistimulation in the upper and lower flanks of the rice shoot base provided a spatial resolution of gravity-induced transcriptomic changes.

MapMan and PageMan analyses provide a systematic description of the attributes of gene products [48], [49], [50], [51]. In comparing our results in rice with the profiles of *Arabidopsis* gravitropism responsive genes, many transcripts were found to be altered by gravistimulation in both plants. These include the genes transcripts coding for cytochrome P450, expansin, xyloglucan endotransglycosylases, pectinesterases, as well as auxin-responsive genes (*SAUR*, *IAAs*, auxin and efflux carrier *EIR1* or *PIN2*) [24], [26]. Some common transcripts have also been identified between *Brassica* and rice which are changed asmmetrically by gravistimulation between the upper and lower flanks at the bending site (e.g., expansin family genes, *SAUR*, *IAAs* and glycosyl related genes) [27]. These results suggest some similarities in the transcriptional changes caused by gravistimulation between monocotyledons and dicotyledons. Our data showed that transcripts involved in the synthesis of ethylene and cytokinin, as well as secondary metabolism (including synthesis of bioflavonoids and phenylpropanoids) were strongly regulated at 0.5 h; meanwhile, transcripts related to hormone metabolism and transportation (e.g., auxin, GA and BR) and cell wall expansion, which may contribute to the asymmetric growth, were differentially altered at 6 h. In addition, JA, glycosis and TCA cycle related genes were induced at both 0.5 h and 6 h.

The TCA cycle is composed of a series of biochemical reactions used by all aerobic organisms to generate energy through oxidization of acetate derived from carbohydrates, fats and proteins into carbon dioxide and water. These reactions provide precursors for the biosynthesis of compounds, including certain amino acids as well as the reducing agent NADH and GTP (or ATP); therefore, the increase in these factors due to elevation of TCA cycle related gene transcripts and corresponding proteins may accelerate growth in the lower half flank in the gravitropic response. The important roles of plant hormones (e.g., IAA, GA and ethylene) in gravitropism have been reported in previous studies [22], [23], [52], [53], [54], [55]. Here, we systematically identified transcripts involved in the homeostasis of plant hormones, especially GA and JA. The content of bioactive GAs was found to be increased in the lower flank of the rice shoot base, implicating the involvement of GA in gravitropism. However, the mechanistic basis of asymmetric distribution of bioactive GAs has not been clearly defined [21]. In this study, we observed increased expression of *OsGA20ox4*, the product of which synthesizes bioactive GAs and decreased expression of *OsGAox7* and *OsGA2ox9*, which are involved in the degradation of bioactive GAs in the lower flank of the rice shoot base. This result suggested that differences in the abundance of these transcripts between the upper and lower flanks of the shoot base may be responsible for the asymmetric distribution of bioactive GAs in gravitropism. Consistent with this observation, EUI, a cytochrome P450 monooxygenase which deactivates bioactive GAs, was reported previously to play a role in root gravitropism in rice [23]. In addition, we found that transcripts involved in JA biosynthesis and distribution were asymmetrically expressed in the two flanks of the rice shoot base. Gutjahr *et al.* (2005) detected a gradient of JA opposing the auxin gradient related to gravitropism in rice [39]. Few genes in JA homeostasis have been reported in gravitropism research. In this work, three JA biosynthesis related gene transcripts encoding lipoxygenase 1 (Os03g49260), cytochrome P450 (Os02g12690) and 12-oxophytodienoate reductase 2 (Os06g11200) were identified to be less abundant in the lower flank of the rice shoot base. This asymmetric transcriptional pattern may have affected the JA gradient across the upper and lower flank of the rice shoot base.

In summary, we have identified a series of biological processes and related gene transcripts in gravitropism by transcriptional analysis of rice shoot bases after gravistimulation. Functions of these gene products were analyzed by MapMan and PageMan to identify the key genes in the plant response to gravity. Our study provides important clues and candidate genes for future research to experimentally validate their involvement in gravitropism.

## Methods

### Plant materials and gravitropic stimulation

Rice plants (*Oryza sativa* sp. japonica) were germinated and grown in soil at a photon flux density of 350–400 µmol m^−2^ s^−1^, 60–80% relative humidity and 12/12 h day/night cycle at 25°C in a phytotron. All experiments were performed with 3-week-old seedlings. The two flanks of the rice shoot base between 0.5 cm and 1 cm above the root were marked (Fig. S6), and seedlings were kept in the dark for 16 h prior to gravistimulation. Some plants were harvested as 0 h samples and stored in liquid nitrogen, while other plants were subsequently gravistimulated by a 90° rotation to the horizontal position. The entire treatment process was carried out under dim green light. At 0.5 h and 6 h after the reorientation, the whole plants were quickly pulled from the soil and frozen in liquid nitrogen. The lower and upper flanks of the rice shoot base (exactly between 0.5 cm and 1 cm above the root) were separated rapidly on glass panes on ice by cutting with a razor blade, refreezing in liquid nitrogen and storing at −70°C until RNA extraction. Three biological replicates for each time point were harvested and used for subsequent experiments. Each pooled sample contained either 50 shoot bases of rice that were not reoriented (0 h and 6 h) or 100 lower or upper flanks of shoot bases (0.5 h upper, 0.5 h lower, 6 h upper and 6 h lower) from plants grown in the same condition.

### RNA isolation, microarray hybridization and analysis

Total RNA was extracted using QIAGEN RNAeasy mini kit (Qiagen, Valencia, CA) according to the manufacturer's instructions. Quality and integrity of the isolated RNA were assessed on the Agilent 2100 bioanalyzer (Agilent Technologies, Palo Alto, CA). Total RNA was incubated with oligo dT/T7 primers and reverse transcribed into double-stranded cDNA. *In vitro* transcription of the purified cDNA was performed with T7 RNA polymerase at 42°C for 6 h. The amplified RNA was purified and subjected to a second round of amplification and biotin labeling with the Affymetrix IVT labeling kit. Biotin-labeled RNA was fragmented and hybridized to Affymetrix Rice Genome GeneChips for 16 h, washed, stained and scanned. GCOSv1.4 was used to calculate the signal intensity and the calls on the hybridized GeneChip. Signal intensities were then centered to the 50th percentile of each chip and for each individual gene. Only genes labeled by the GCOS software as “present” or “marginal” in all samples were included in further analysis. We filtered the changed transcripts according at different times by RVM ANOVA [56], [57]. The Benjamini-Hochberg *P*-value correction (*P*-value cut-off 0.05) was used to recognize significant differences in transcript abundance. Heat maps were generated by using the Matrix2png online software [58].

### qRT-PCR analysis

For qRT-PCR analysis, cDNA was synthesized using oligo (dT) 18 primers and ReverTra Ace M-MLV RTase (Toyobo, Japan) using total RNA as template according to the manufacturer's recommendations. Primers used for qRT-PCR are listed in Table S4. qRT-PCR was performed with the SYBR Green Real-time Master Mix (Toyobo), and fluorescent emission was detected with a Chromo4 Four-Color Real-Time PCR System (MJ Research, San Francisco, CA). *Ubiquitin 5* (*OsUBQ5*, Os01g22490) was used as the reference gene. The relative gene expression level was calculated as the ratio of target genes over *UBQ5* using a comparative cycle threshold (Ct) method [2].

### MapMan and PageMan analyses

MapMan [29], [30] and PageMan [29] analyses were performed using a set of unique probe sets which were obtained from the Gene Expression Omnibus (GEO) database at the National Center for Biotechnology Information (NCBI) under the series accession number GSE31834, and all data deposited into GEO were MIAME (minimum information about a microarray experiment) compliant. To identify those BINs that were significantly affected by gravitropism, we calculated the induction factor of all genes in a BIN and compared the average induction factor of a BIN to that of all other BINs by the Wilcoxon rank sum test. Annotations of genes without references in the manuscript all corresponded to related orthologous genes in the database.

## Supporting Information

Figure S1 MapMan and PageMan analyses for significantly changed transcripts in the lower and upper flanks at 0.5 h and 6 h after gravistimulation. Significant fold changes in transcript levels between samples were log transformed and analyzed using the PageMan tool. Wilcoxon statistical analysis with Benjamini-Hochberg false discovery rate control was performed to determine significantly different gene categories. Non-significant categories were collapsed for display. Statistically significant differences are represented by a false color heat map (blue  =  up-regulated; red  =  down-regulated), where a z-score of 1.96 represents a false discovery rate-corrected *P* value of 0.05.
(TIF)

Figure S2 MapMan and PageMan analyses for changed transcripts in lower and upper flanks at 0.5 h and 6 h after gravistimulation compared to the control sample at 0 h. Significant fold changes in transcript levels between samples were log transformed and analyzed using the PageMan tool. Wilcoxon statistical analysis with Benjamini-Hochberg false discovery rate control was performed to determine significantly different gene categories. Non-significant categories were collapsed for display. Statistically significant differences are represented by a false color heat map (red  =  up-regulated; blue  =  down-regulated), where a z-score of 1.96 represents a false discovery rate-corrected *P* value of 0.05.
(TIF)

Figure S3 Changes in transcript abundance of cytokinin and ethylene related genes at 0.5 h after gravistimulation. **A.** Visualization of modulated transcripts in the cytokinin pathway: lower at 0.5 h vs. upper at 6 h. **B.** Visualization of the modulated transcripts in the ethylene pathway: lower flank at 0.5 h vs. upper flank at 0.5 h. Significant fold changes in transcripts were log transformed (red, up-regulated; blue, down-regulated).
(TIF)

Figure S4 Heat map of transcripts changed only at 0.5 h after gravistimulation. Significant fold changes in transcript levels between samples were log transformed. Wilcoxon statistical analysis with Benjamini-Hochberg false discovery rate control was performed to determine significantly different gene categories. Statistically significant differences are represented by a false color heat map (red  =  up-regulated; green  =  down-regulated).
(PNG)

Figure S5 Heat map of transcripts altered only at 6 h after gravistimulation. Significant fold changes in transcript levels between samples were log transformed. Wilcoxon statistical analysis with Benjamini-Hochberg false discovery rate control was performed to determine significantly changed transcript categories. Statistically significant differences are represented by a false color heat map (red, up-regulated; green, down-regulated).
(PNG)

Figure S6 Illustration of sample harvest. The short base of rice in the frame was harvested in our experiments, and the arrow indicates the gravity direction. The rice shoot base was divided into the upper and lower flank along the midline indicated by the white line.
(TIF)

Table S1 Annotated list transcripts altered both at 0.5 h and 6 h after horizontal reorientation. Significant fold changes in transcripts were log transformed.
(XLS)

Table S2 Annotated list of transcripts altered only at 0.5 h after gravistimulation. Significant fold changes in transcripts were log transformed.
(XLS)

Table S3 Annotated list of transcripts altered only at 6 h after gravistimulation. Significant fold changes in transcripts were log transformed.
(XLS)

Table S4 Primers used for qRT-PCR analysis.
(TIF)
