# Supplementary figures and images for: Microarray Analyses and Comparisons of Upper or Lower Flanks of Rice Shoot Base Preceding Gravitropic Bending
Source: PLoS One. 2013 Sep 5;8(9):e74646. doi: 10.1371/journal.pone.0074646 (PMC3764065; doi:10.1371/journal.pone.0074646)

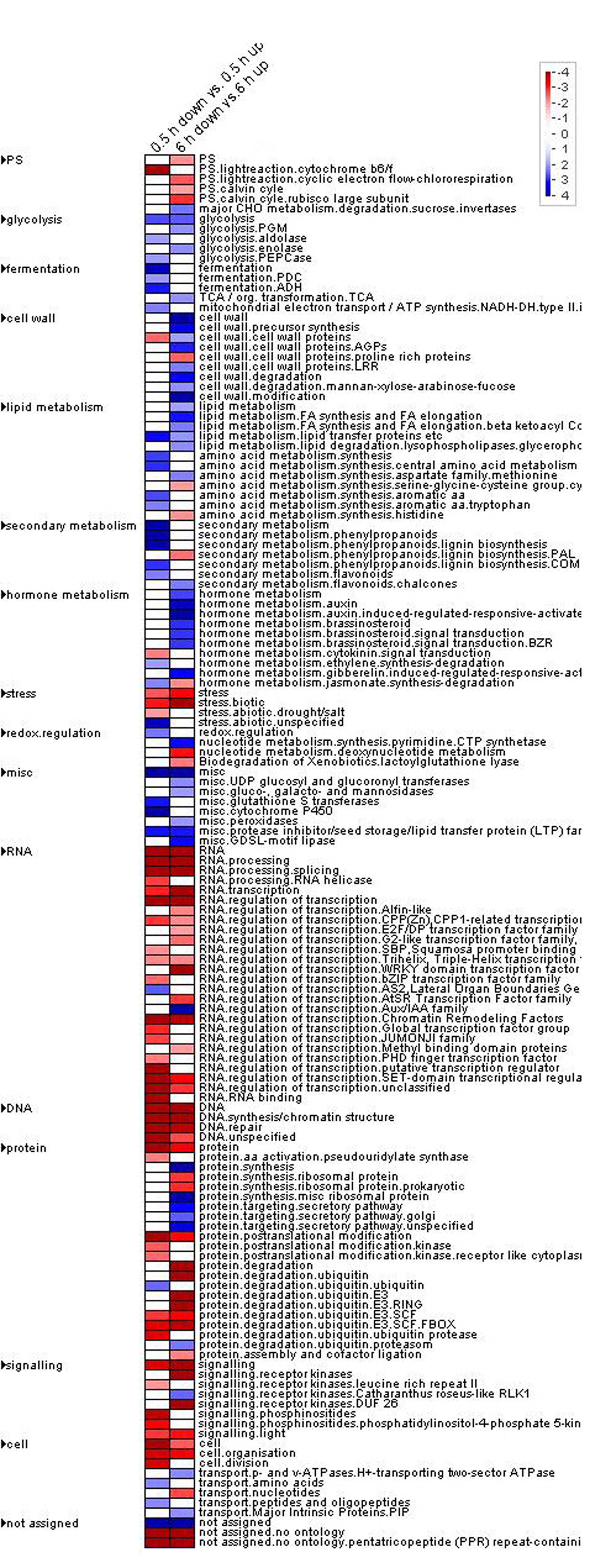

Supplement: Figure S1 — MapMan and PageMan analyses for significantly changed transcripts in the lower and upper flanks at 0.5 h and 6 h after gravistimulation. Significant fold changes in transcript levels between samples were log transformed and analyzed using the PageMan tool. Wilcoxon statistical analysis with Benjamini-Hochberg false discovery rate control was performed to determine significantly different gene categories. Non-significant categories were collapsed for display. Statistically significant differences are represented by a false color heat map (blue = up-regulated; red = down-regulated), where a z-score of 1.96 represents a false discovery rate-corrected P value of 0.05. (TIF) [file pone.0074646.s001.tif]

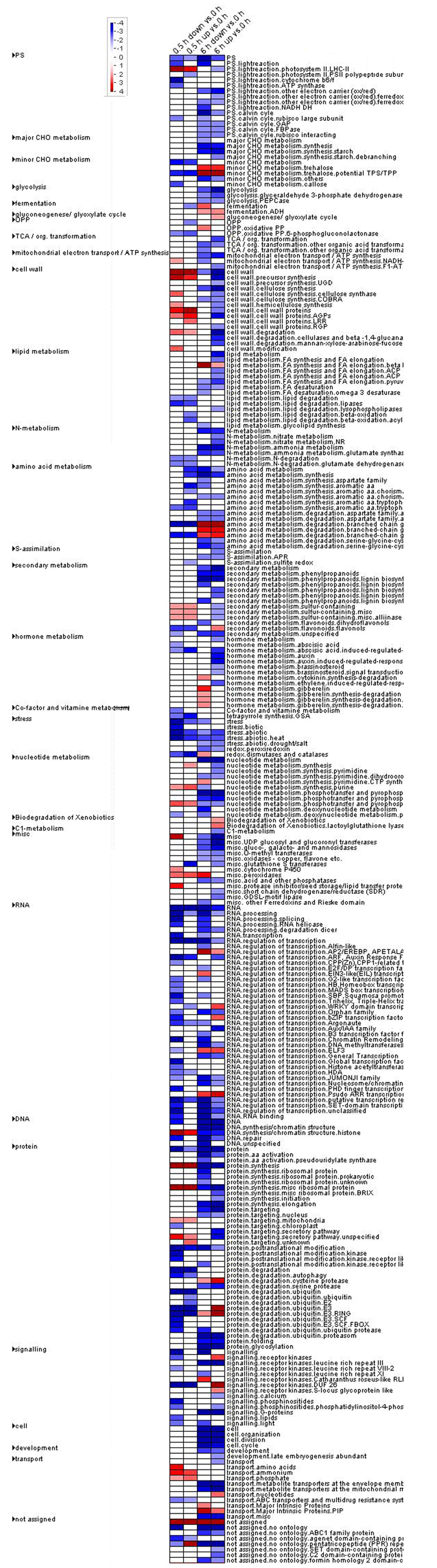

Supplement: Figure S2 — MapMan and PageMan analyses for changed transcripts in lower and upper flanks at 0.5 h and 6 h after gravistimulation compared to the control sample at 0 h. Significant fold changes in transcript levels between samples were log transformed and analyzed using the PageMan tool. Wilcoxon statistical analysis with Benjamini-Hochberg false discovery rate control was performed to determine significantly different gene categories. Non-significant categories were collapsed for display. Statistically significant differences are represented by a false color heat map (red = up-regulated; blue = down-regulated), where a z-score of 1.96 represents a false discovery rate-corrected P value of 0.05. (TIF) [file pone.0074646.s002.tif]

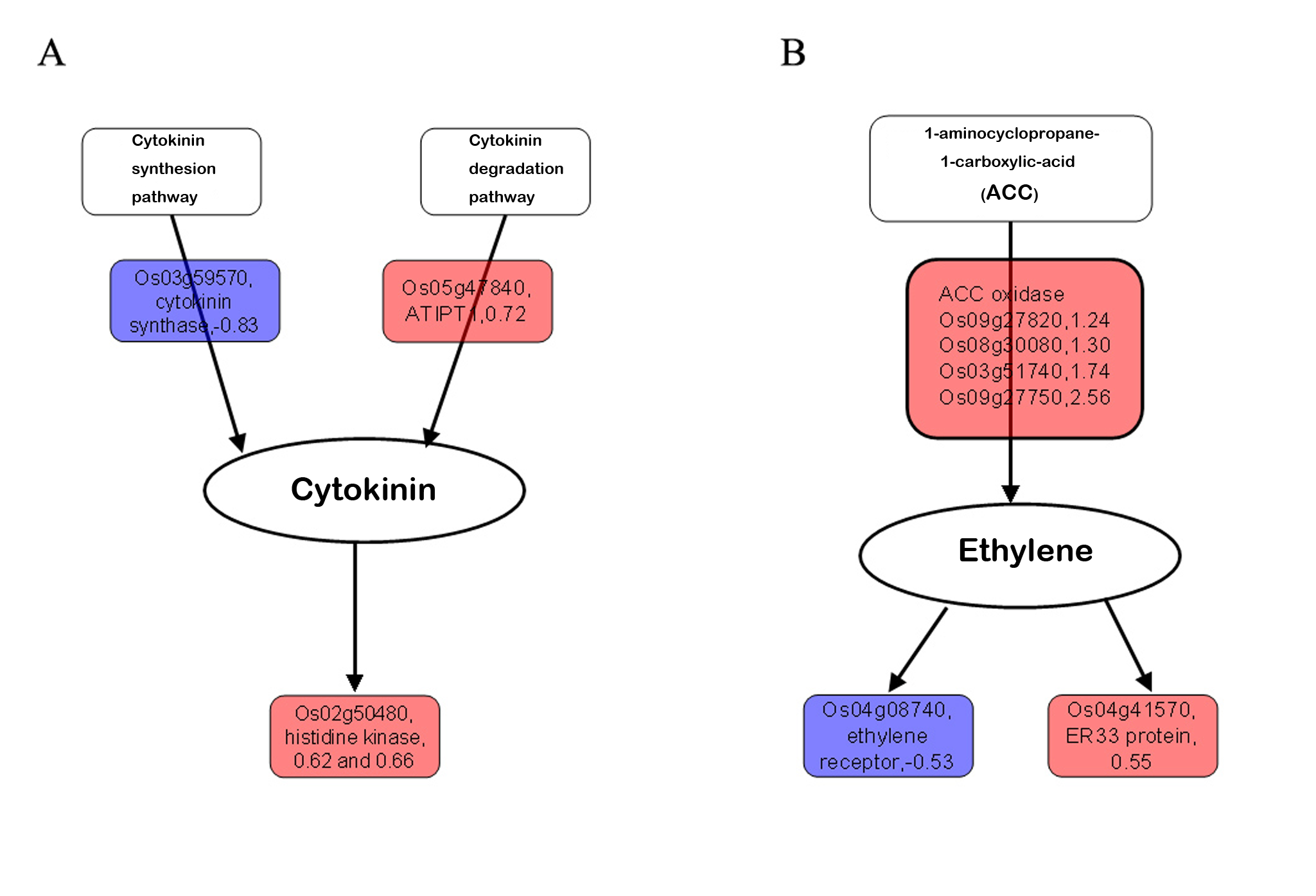

Supplement: Figure S3 — Changes in transcript abundance of cytokinin and ethylene related genes at 0.5 h after gravistimulation. A. Visualization of modulated transcripts in the cytokinin pathway: lower at 0.5 h vs. upper at 6 h. B. Visualization of the modulated transcripts in the ethylene pathway: lower flank at 0.5 h vs. upper flank at 0.5 h. Significant fold changes in transcripts were log transformed (red, up-regulated; blue, down-regulated). (TIF) [file pone.0074646.s003.tif]

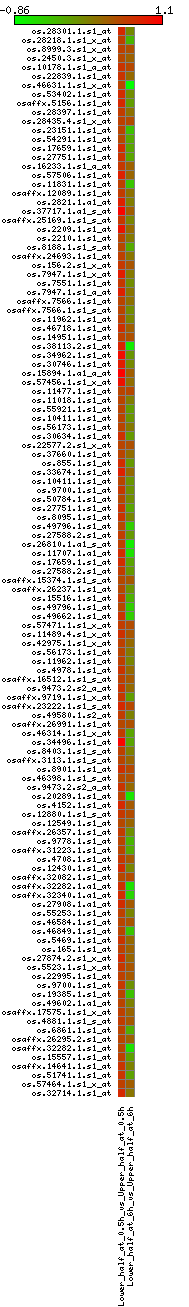

Supplement: Figure S4 — Heat map of transcripts changed only at 0.5 h after gravistimulation. Significant fold changes in transcript levels between samples were log transformed. Wilcoxon statistical analysis with Benjamini-Hochberg false discovery rate control was performed to determine significantly different gene categories. Statistically significant differences are represented by a false color heat map (red = up-regulated; green = down-regulated). (PNG) [file pone.0074646.s004.png]

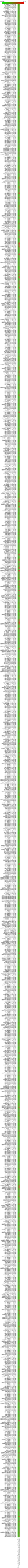

Supplement: Figure S5 — Heat map of transcripts altered only at 6 h after gravistimulation. Significant fold changes in transcript levels between samples were log transformed. Wilcoxon statistical analysis with Benjamini-Hochberg false discovery rate control was performed to determine significantly changed transcript categories. Statistically significant differences are represented by a false color heat map (red, up-regulated; green, down-regulated). (PNG) [file pone.0074646.s005.png]

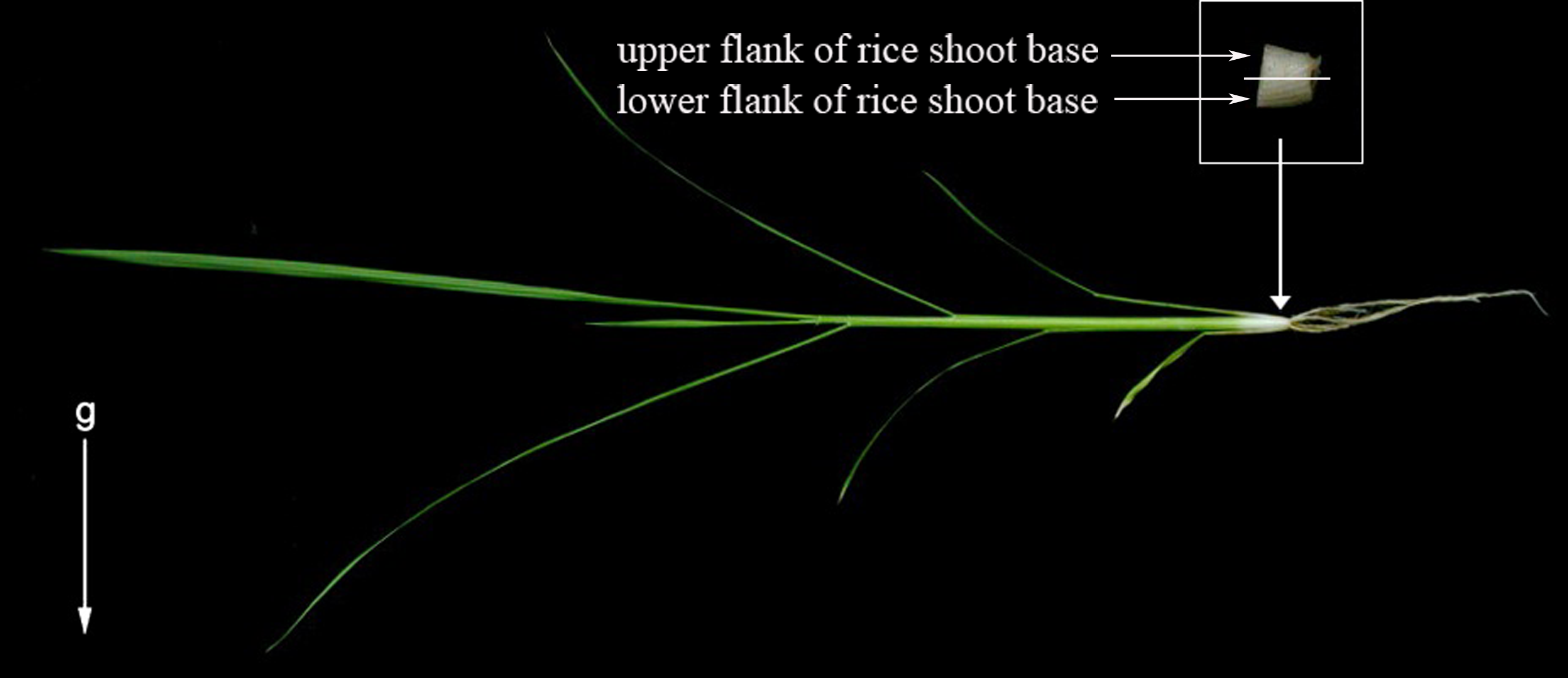

Supplement: Figure S6 — Illustration of sample harvest. The short base of rice in the frame was harvested in our experiments, and the arrow indicates the gravity direction. The rice shoot base was divided into the upper and lower flank along the midline indicated by the white line. (TIF) [file pone.0074646.s006.tif]

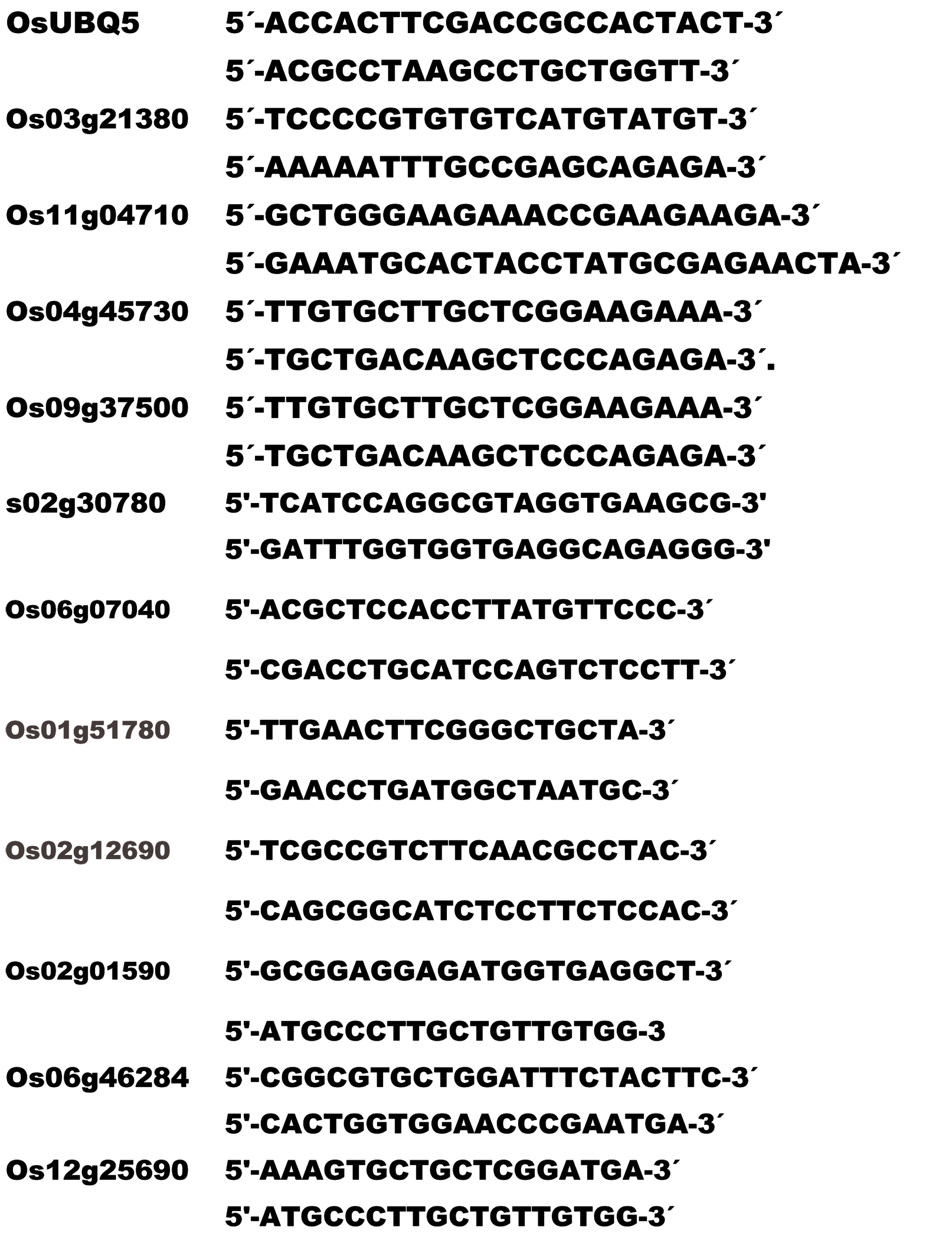

Supplement: Table S4 — Primers used for qRT-PCR analysis. (TIF) [file pone.0074646.s010.tif]
